# Identification of circulating human papillomavirus types through high-throughput sequencing of Canadian municipal and institutional wastewater samples

**DOI:** 10.1128/aem.00348-25

**Published:** 2025-06-05

**Authors:** Shayna J. Giesbrecht, Samantha J. Krosta, Rebecca Fox, Kurt Kolsun, Zoe Quill, Suzanne Gibbons, Aida Sivro, Paul Sandstrom, Chand S. Mangat, Michael G. Becker

**Affiliations:** 1 Department of Microbiology, University of Manitoba 468335, Winnipeg, Manitoba, Canada; 2 JC Wilt Infectious Diseases Research Centre, National Microbiology Laboratory Branch, Public Health Agency of Canada, Winnipeg, Manitoba, Canada; Centers for Disease Control and Prevention, Atlanta, Georgia, USA

**Keywords:** wastewater, human papillomavirus, HPV, wastewater-based epidemiology, surveillance studies, wastewater-based testing, Canada, correctional facility, sequencing

## Abstract

Human papillomaviruses (HPVs) are a diverse group of viruses that infect human mucosal and cutaneous tissues, with a ubiquitous global distribution. Several types are classified as high-risk due to their association with cervical, anal, penile, and oropharyngeal cancers. Given its widespread prevalence and the limitations of clinical surveillance, HPV is an ideal candidate for wastewater-based testing (WBT), as a way to characterize circulating HPV types. Toward this goal, we utilized high-throughput Illumina-based amplicon sequencing targeting the conserved GP5+/GP6+ sites of HPV in wastewater samples collected from two urban treatment plants, two town sites, and three correctional facilities. Larger population catchments displayed greater diversity of HPV types in wastewater compared to institutional settings, reflecting differences in community composition and exposure. A total of 24 distinct HPV types were detected, all belonging to the alpha-papillomavirus genus, encompassing high- and low-risk types, types of unknown risk, and both cutaneous and mucosal types. Among these were six vaccine-preventable types, including HPV-16 and HPV-18, as well as probably carcinogenic types not routinely monitored in clinical settings, such as HPV-67, HPV-69, and HPV-73. These findings demonstrate the utility of WBT as a complementary tool to clinical testing, providing critical insights to inform cancer prevention strategies and evaluate the impact of vaccination programs.

**IMPORTANCE:**

Human papillomavirus (HPV)-related cancers, including cervical, anal, penile, and oropharyngeal cancers, impose a significant economic burden on healthcare systems, with billions spent annually on treatment and management. This study reports the detection of HPV in Canadian wastewater, providing new insights into HPV circulation within communities. The findings highlight the ongoing prevalence of high-risk HPV types targeted by vaccines and the circulation of probable carcinogenic types, such as HPV-67, HPV-69, and HPV-73, which are not included in current clinical testing algorithms. Wastewater-based surveillance of HPV could complement clinical testing by capturing data on populations typically underrepresented in clinical settings. A clearer understanding of circulating HPV types can support public health efforts to promote cancer screening, monitor vaccination campaigns, and address gaps in prevention strategies. The continued development of wastewater-based testing systems hints toward a promising future for unbiased monitoring of community health.

## INTRODUCTION

Wastewater-based testing (WBT) involves analyzing sewage influent for markers relevant to public and environmental health. WBT has been used for monitoring infectious disease (1, 2), pharmaceutical and illicit drug usage (3–6), industrial chemical contamination (7), and antimicrobial resistance (8, 9). A major advantage of WBT is that it provides a comprehensive assessment of community health, encompassing all individuals contributing to the sewage system. Specifically, for infectious disease surveillance, WBT is a valuable tool for confirming the presence of an infectious disease within a community and tracking disease transmission trends (2, 10, 11).

The value of WBT stems from its inclusivity, capturing data from those unable or unlikely to present for clinical testing, including asymptomatic individuals, as well as those experiencing barriers to accessing the medical system (12, 13). Gaps in clinical COVID-19 surveillance led to the establishment of national and municipal wastewater testing programs globally (14–20). Following its success in tracking severe acute respiratory syndrome coronavirus 2 (SARS-CoV-2), WBT has been rapidly adapted for other pathogens, including influenza (21–25), respiratory syncytial virus (21, 24, 26), mpox (27–31), and dengue virus (32), and efficiency has been improved through multiplexing for simultaneous detection of multiple pathogens (33, 34). Genome sequencing from wastewater has also been particularly useful for tracking SARS-CoV-2 mutations (25, 35–37).

The case for incorporating human papillomavirus (HPV) into WBT programs is strong, given its widespread distribution and the limited scope of clinical surveillance approaches. HPV is a non-enveloped, double-stranded, circular DNA virus belonging to the *Papillomaviridae* family. The HPV genome encodes eight open reading frames: the early genes (E1, E2, E4, E5, E6, and E7) and the late genes (L1 and L2) (38, 39). The early genes encode proteins that facilitate replication, while late genes encode structural proteins (38, 39). Studies agree that the mutation rate of HPV is low, with estimates ranging from 2.2 × 10^−9^ to 4.5 × 10^−7^ per site per year (40–42). The L1 gene has the lowest mutation rate of all HPV genes (43) and is the most conserved region of the genome (43, 44). Due to its highly conserved nature, HPV types are classified based on the sequence of the L1 gene (38). To be considered a distinct type, the DNA sequence of the L1 gene must have a difference of greater than 10% compared to the closest known type (38). Currently, there are more than 150 HPV types that have been classified by the Papillomavirus Episteme, with many more awaiting acceptance (45). HPV types can be subdivided into five broad genera: alpha-papillomavirus, beta-papillomavirus, mu-papillomavirus, nu-papillomavirus, and gamma-papillomavirus.

HPV infection can vary in manifestation, ranging from benign cutaneous lesions to high-grade cervical intraepithelial neoplasms, and is associated with various cancers in both males and females (46). Cutaneous HPVs are typically associated with benign skin warts (e.g., HPV1 and HPV2). Benign cutaneous lesions and skin warts can be caused by types within the alpha-papillomavirus, beta-papillomavirus, mu-papillomavirus, nu-papillomavirus, and gamma-papillomavirus genera (38). Most cutaneous types are not associated with cancers, though certain β-genus HPVs have been implicated as co-factors in non-melanoma skin cancers under conditions like chronic UV exposure (47). HPV types associated with genital and mucosal tissues are part of the alpha-papillomavirus genus and can be further categorized by oncogenic risk. Low-risk types (types 6, 11, 40, 42, 43, 44, 54, 61, 72, 74, 81, 83, 84, 86, 87, 90, and 91) cause benign lesions such as genital warts, whereas high-risk or possibly high-risk types (types 16, 18, 31, 33, 35, 39, 45, 51, 52, 56, 58, 59, 66, 68, 70, 73, and 82) have the potential to induce high-grade lesions and cancerous changes to cells (48–52).

Cervical cancer is the fourth most common cancer in biological females worldwide (53), with high-risk HPV (HR-HPVs) types implicated in nearly 100% of cervical cancers (54) and HPV-16 and HPV-18 responsible for 50–70% of all cases (55, 56). Additionally, HPV has been heavily implicated in other cancers. HPV is estimated to be the cause of approximately 90% of anal cancers (57), 60% of penile cancers, and up to 60–70% of oropharyngeal cancers (46)—primarily through host integration and upregulation of E6 and E7 proteins (39). The E6 and E7 genes inhibit and degrade host factors controlling cell proliferation and apoptosis, effectively immortalizing cells and causing chromosomal instability and uncontrolled cell proliferation (58). Cancers linked to HR-HPV remain a significant concern globally, necessitating efforts to target HR-HPVs for prevention. In the United States alone, healthcare costs associated with HPV are estimated to be over nine billion dollars annually (59).

Studies estimate that 70–85% of adults will acquire at least one HPV infection in their lifetime, the majority of which will be asymptomatic (60, 61). HPV takes between 7 and 20 years on average to progress to precancerous lesions termed high-grade cervical intraepithelial neoplasia (62, 63), making effective screening an important component of preventing cervical cancer. In Canada, the age-related incidence and mortality of cervical cancers have decreased since the 1970s due to improved prevention and screening (64, 65), including the Papanicolaou test, colloquially the Pap smear, as well as the approval of the Gardasil 4 (66), Cervarix (67), and Gardasil 9 vaccines (68). The Gardasil 4 vaccine, which covers types 6, 11, 16, and 18, was approved in Canada in 2007 (66). Since then, two additional vaccines have been approved in Canada: Cervarix in 2010, which covers types 16 and 18 (67), and Gardasil 9 in 2015, which covers types 6, 11, 16, 18, 31, 33, 45, 52, 58 (68). Data on HPV infections are not systematically recorded in Canada; thus, the true incidence and distribution of types are unknown. Many HPV types are shed in urine (69–71) and stool (72). Notably, virome (73, 74) and targeted sequencing (75, 76) have detected HPV in wastewater samples, suggesting they harbor valuable information about the population-level distribution and prevalence of HPV types.

This study aims to develop a cost-efficient, high-throughput, amplicon-based next generation sequencing (NGS) approach to monitor community levels of circulating HPV subtypes in wastewater. Ultimately, through this approach, we seek to improve the understanding of the HPV landscape, in support of targeted public health interventions and effective resource allocation.

## MATERIALS AND METHODS

### Sample collection

Composite primary influent samples were collected over a 24–48 hour period from two Canadian metropolitan areas with populations greater than 100,000 residents (Winnipeg and Regina), two municipalities with less than 20,000 residents (Town Site 1 in Ontario; Town Site 2 in Manitoba), and three Canadian correctional facilities housing 600–800 long-term residents. At Correctional Facility 1 exclusively, grab samples were collected at peak flow due to limitations in site infrastructure. Samples were collected into sterile polyethylene terephthalate bottles and shipped on ice to the JC Wilt Infectious Diseases Research Centre in Winnipeg, where they were immediately stored at 4°C and tested within 72 hours. Samples were collected between March 2023 and August 2023, with disruptions in April 2023 due to unforeseen labor disruptions at correctional facilities.

### Nucleic acid extraction

For each sample, 100 mL of wastewater was clarified by centrifugation at 4,200 × *g* for 50 minutes at 4°C. The resulting pellet was resuspended in 650 µL of warmed Solution MBL with 2.5% 2-mercaptoethanol and 100 µL of 25:24:1 phenol:chloroform:isoamyl alcohol in a PowerBead Pro tube (Qiagen). The mixture was homogenized using a Bead Mill 24 (Thermo Fisher Scientific) for six 30-second cycles at 5 m/s, with 5-second rests between cycles. After homogenization, the sample was centrifuged at 15,000 × *g* for 3 minutes, and the supernatant was extracted following the manufacturer’s protocol for the KingFisher Flex System (Thermo Fisher Scientific), with 2 mg of carrier RNA added prior to extraction.

### Target DNA amplification

A 151 bp region (variable by type) of the conserved HPV L1 gene was amplified with GP5+/GP6+ primers (full list in Table S1; [77]). For each reaction, conducted in triplicate, 8 µL of extract was used, with a reaction volume of 50 µL and concentrations of 600 nM for each primer (Integrated DNA Technologies), 4 nM MgCl_2_, and 200 nM deoxynucleotide triphosphates (dNTPs; Invitrogen Life Technologies) using Invitrogen Platinum Taq DNA Polymerase Master Mix (Invitrogen Life Technologies). PCR was performed under the following conditions: 94°C for 5 minutes; followed by 45 cycles of 94°C for 30 seconds, 50°C for 1 minute, and 72°C for 30 seconds; followed by a final extension of 7 minutes at 72°C. The L1 primers were designed to include Illumina sequencing adaptors and unique seven-nucleotide barcodes (indices) to facilitate direct sequencing of amplicon and pooled sequencing (Table 1).

**TABLE 1:** Primer sequences used in this study, including Illumina i5 and i7 adapters, sequencing primer sites, a unique sample barcode, and GP5+/GP6+ primer sites

	Primer sequence (5′ → 3′)
Forward primer	*AATGATACGGCGACCACCGAGATCTACACTCTTTCCCTACACGACGCTCTTC* *CGATCT* **GGTTTGTTACTGTGGTAGATACTAC**
Reverse primer	*CAAGCAGAAGACGGCATACGAGAT* NNNNNNN*GTGACTGGAGTTCAGACGT* *GTGCTCTTCCGAT* **CTGAAAAATAAACTGTAAATCATATTC** ^*a*^

^*a*^ NNNNNNN replaced with unique 7-nucleotide barcode as summarized in Table S1. Italic text indicates Illumina i5 and i7 sequencing adaptors, italic underlined text indicates Illumina sequencing primer sites, underlined bolded text indicates two-nucleotide spacer, underlined sequence indicates unique sample barcode (index), and bold text indicates sequence-specific GP5+/GP6+ primer sequences.

As a positive control, plasmids containing the sequences for the L1 gene were amplified for multiple HR-HPV types (types 16, 18, 31, 33, 35, 39, 45, 51, 52, 56, 58, 59, 66, and 68 as validated by Zubach et al. [78]). The plasmids were prepared at concentrations ranging from 2,000 to 5,000 copies/mL and were tested in triplicate.

### Illumina sequencing and analysis

PCR cleanup was performed using AMPure XP (Beckman Coulter) bead-based purification at a bead-to-template ratio of 1.0 as per the manufacturer’s instructions. Product size and concentration were assessed using the Agilent 2200 TapeStation (Agilent) and Qubit Flex Fluorometer (Thermo Fisher Scientific). Samples were pooled to an equimolar concentration, and 150 bp paired-end sequencing was performed using the Illumina MiSeq Reagent Kit v2 kit with a 10–15% PhiX spike-in to increase library complexity.

The MiSeq Control Software removed adapters and separated reads based on their unique barcode. Primer removal and quality filtering were performed using Trimmomatic v0.39 (79), with a sliding window of 5 bp and quality threshold of 15, as well as exclusion of sequences with an average *Q*-score below 30. Paired sequences were assembled and merged using PEAR (80). Trimmed reads were mapped using the NCBI BLAST+ package (81) against a custom, local database of HPV reference genomes derived from the Papillomavirus Episteme (45). Based on optimization to reduce background based on the sequencing of our reference plasmids, alignments used an *e*-score cut-off of 0.001, a minimum percent identity of 93%, and required ≥80 nucleotide identities between the query and aligned reference sequence. The relative abundance of each HPV type was calculated by dividing the number of reads matching a particular HPV type by the total reads matching HPV in that sample. Any HPV types representing <0.2% of the total sample composition were removed from subsequent analysis. Data were visualized in R Studio version 4.4.2 (82) using the package ggplot2 (83). To assess the sample diversity, Shannon’s Diversity Index was calculated for each sample, and the results were averaged for each site.

## RESULTS

### Overview of sequencing results

In total, 86 samples were sequenced across this study. Nine samples failed to produce reads, and an additional four samples failed to meet quality control metrics (outside of acceptable *Q*-scores or *e*-scores). More than 18 million paired reads were generated, with 50.5% of reads passing filter, for an average of 237,531 (95% CI 137,739–337,323) reads per successful sample. Of these reads, a total of 11,148,715 passed quality filtering and successfully aligned to an HPV type (Table S2), with the vast majority of unaligned reads identified as PhiX spike-in controls.

**Fig 1:**
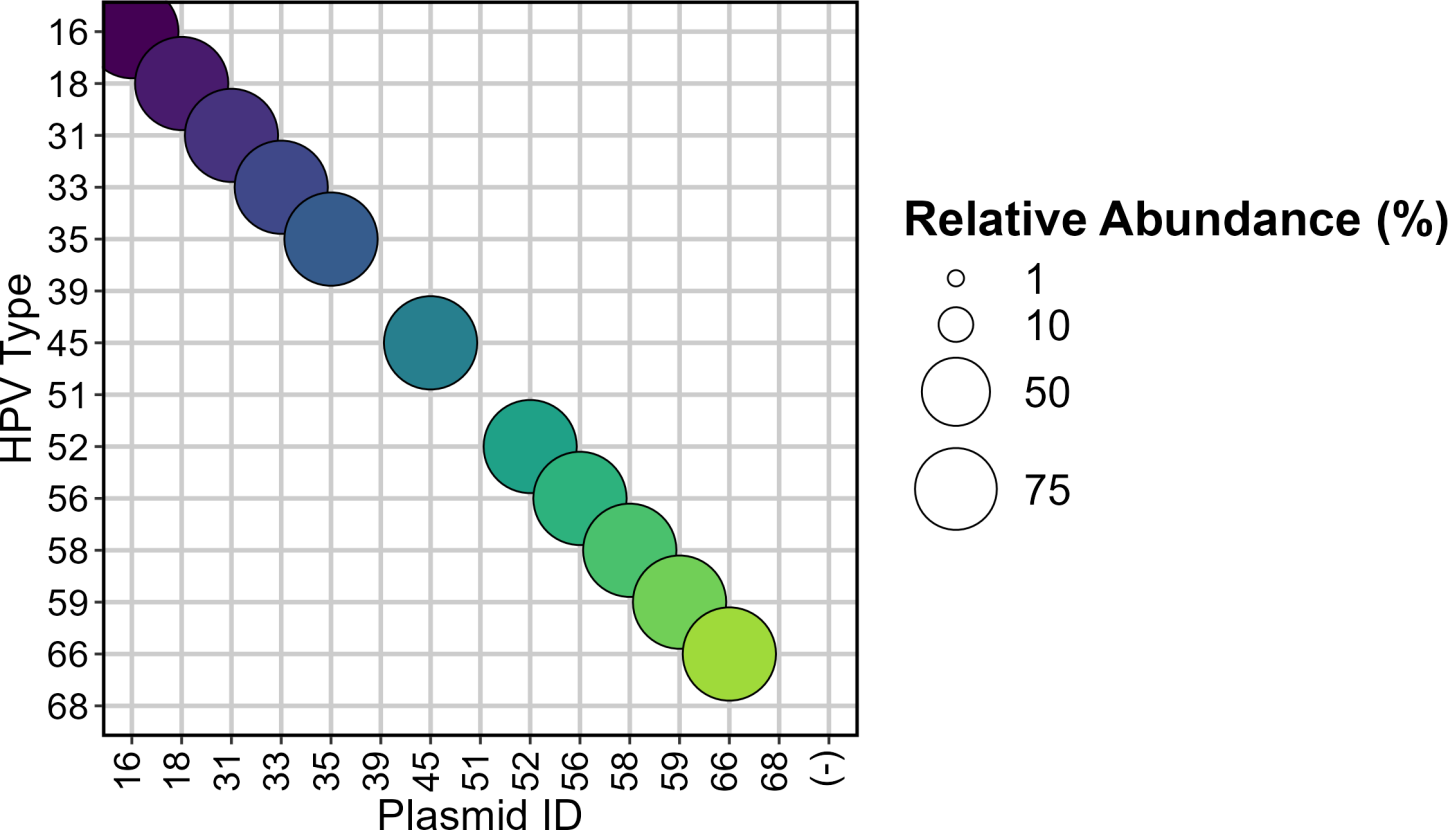
Relative abundance plot of HPV types detected from plasmids containing the L1 gene of HPV (*x*-axis). Bubble size corresponds to the relative abundance of the HPV type. Negative control with no plasmid (−) contains only nuclease-free water. Three plasmids, HPV-39, HPV-51, and HPV-68, failed to sequence. Colorization is used to aid in distinguishing HPV types.

Only the alpha-papillomavirus genus was detected among the 24 types observed in wastewater samples in this study. These included high- and low-risk types, types of unknown risk, and cutaneous and mucosal types. For all reference plasmids that amplified (11/14), 100% of reads matched the appropriate HPV type. The plasmids for HPV-39, HPV-51, and HPV-68 failed to amplify (Fig. 1).

### Type-specific HPV detection in wastewater from Winnipeg, Regina, and town sites

**Fig 2:**
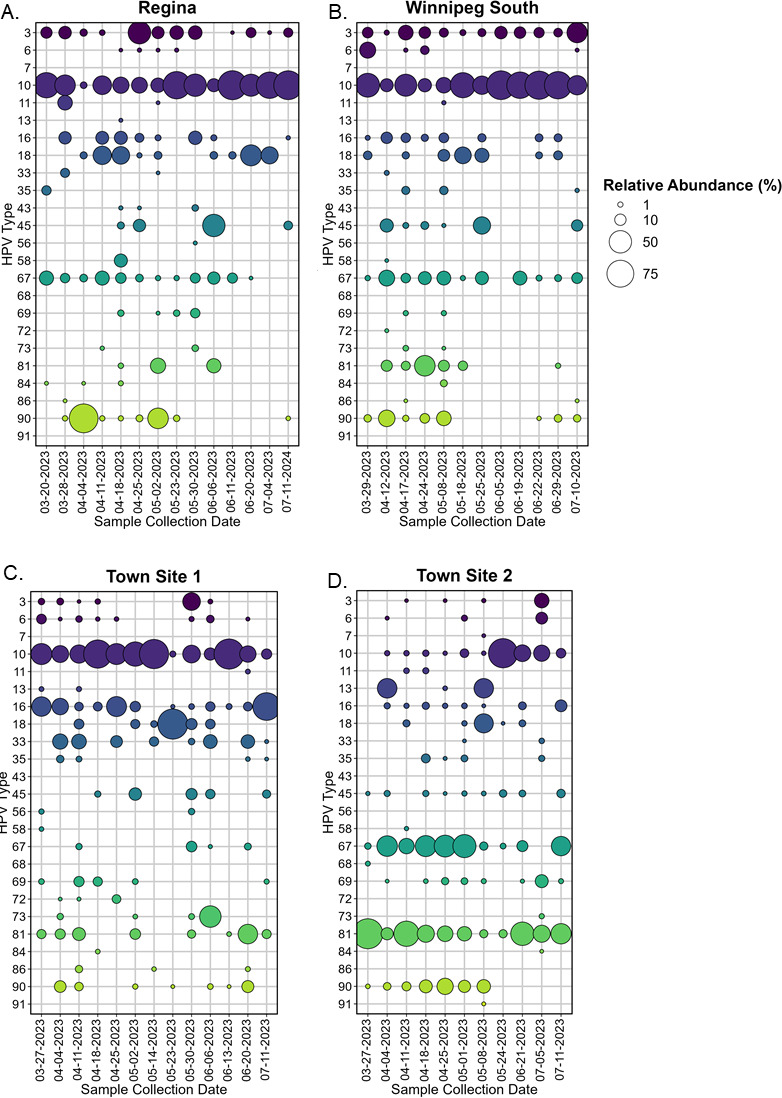
Relative abundance plot of HPV types detected in wastewater samples from Regina (**A**), Winnipeg South (**B**), Town Site 1 located in Ontario (**C**), and Town Site 2 located in Manitoba (**D**). Bubble size corresponds to the relative abundance of the HPV type. Colorization is used to aid in distinguishing HPV types.

Sixteen HPV types were detected at all sites: HPV-3, HPV-6, HPV-10, HPV-16, HPV-18, HPV-33, HPV-35, HPV-43, HPV-45, HPV-58, HPV-67, HPV-69, HPV-73, HPV-81, HPV-84, and HPV-90. Seven HPV types (HPV-33, HPV-35, HPV-56, HPV-58, HPV-72, HPV-73, and HPV-86) were detected in both urban centers and town sites but not in correctional facilities (Fig. 2A through D). In municipal sites, the types that exceeded 50% of reads on at least one occasion included HPV-10, HPV-16, HPV-18, HPV-45, HPV-67, HPV-81, and HPV-90. The detection of HPV-67, HPV-69, and HPV-73 in this study is noteworthy, as they represent types of probable carcinogenic risk that are not captured through routine clinical testing.

**Fig 3:**
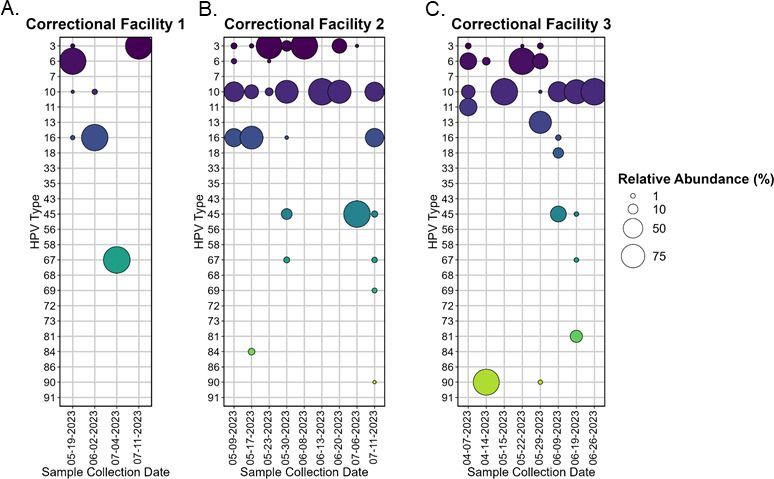
Relative abundance plot of HPV types detected in wastewater samples from three unique Canadian correctional facilities (labeled A-C) housing 600–800 long-term residents. Bubble size corresponds to the relative abundance of the HPV type. Colorization used to aid in distinguishing HPV types.

Two HPV types were detected only once in this study, HPV-7 and HPV-91, which were detected in a sample from Town Site 2 on May 8, 2023 (Fig. 2D). Other infrequently detected types included HPV-43, which was only detected in Town Site 1 (Fig. 2C) and Correctional Facility 3 (Fig. 3C). The average number of HPV types detected in samples collected from municipal sites was 7.7, with a maximum of 14 types detected in a single sample. Shannon’s Diversity Index was similar for all four municipal sites, ranging from 1.10 to 1.26, with Regina and Winnipeg representing the highest and lowest indices, respectively.

### Lower diversity of HPV in wastewater from Canadian correctional facilities

Five HPV types were detected in samples collected from all correctional facilities: HPV-3, HPV-6, HPV-10, HPV-16, and HPV-67. On at least one occasion, the relative abundances of HPV-3, HPV-6, HPV-10, HPV-13, HPV-16, HPV-45, HPV-67, and HPV-90 exceeded 50% (Fig. 3A through C). Overall, there was lower diversity in the number and distribution of HPV types in correctional facilities as compared to municipal sites. For example, 2.8 different HPV types were detected on average in samples from correctional facilities, with a maximum of six types. Further highlighting the lack of diversity in institutional samples, in all but two samples from correctional facilities, more than 85% of reads were characterized as 1–2 different types. Shannon’s Diversity Index indicated lower diversity for all correctional facilities as compared to municipal sites, at 0.05, 0.48, and 0.47 for Correctional Facilities 1, 2, and 3, respectively.

## DISCUSSION

We developed a protocol for WBT of circulating HPV types and applied it across multiple settings, including urban and town sites, and institutional settings. We identified 24 unique HPV types, spanning high-risk, low-risk, and unknown-risk classifications, as well as both cutaneous and mucosal types. We showed that HPV subtypes vary in a regionally specific manner, with sporadic detections of certain types. High-risk HPV-16, HPV-18, HPV-45, HPV-67, and HPV-69 were found at nearly all sites, underscoring their widespread presence despite vaccine availability (50), consistent with previous clinical research indicating the widespread prevalence of HPV-16, HPV-18, and HPV-45 in Canada (51, 84–86). Other high-risk types, including HPV-33, HPV-56, HPV-58, and HPV-72, were detected only at specific locations, highlighting geographic variation. These findings align with previous clinical studies on HPV prevalence in Canada and demonstrate the utility of wastewater testing in monitoring community-level HPV circulation, providing a new tool that can monitor over time the effectiveness of vaccination campaigns or other interventions.

The diversity of HPV types was notably higher in wastewater from larger catchments, suggesting a broad range of HPV exposure and transmission in more densely populated areas. In comparison, samples from correctional facilities exhibited dominance by a few types, likely reflecting more homogenous circulating populations and possibly individual short-term shedding patterns. The transient nature of institutional data may also reflect noise resulting from PCR biases, which may be corrected for in future studies through the application of unique molecular identifiers (87). Unique molecular identifiers allow for the differentiation of each individual DNA molecule preceding or during the early steps of PCR by incorporating a random, unique sequence label (87). This label facilitates the accurate deduplication of reads in downstream bioinformatic processing and absolute measurement of PCR templates (87).

This study also detected a significant presence of cutaneous HPV types (HPV-3, HPV-7, HPV-10), known for causing common warts or flat warts (51, 88). Two of these types, HPV-3 and HPV-10, were consistently found across all sites and often dominated distribution profiles. HPV-3 and HPV-10 are highly contagious through skin-to-skin contact (88), but are of little clinical interest currently as they are not considered carcinogenic (50). Other studies investigating HPV in environmental water sources have recovered a high abundance of cutaneous beta-HPVs (74, 76, 89) that are likely introduced into sewage systems via hand washing and bathing (75, 76). Similar to beta-HPVs, HPV-3 and HPV-10, although alpha-HPVs, are cutaneous and may be released in wastewater in a similar mechanism. Additionally, beta-HPVs have been detected in stool, making this another potential route into sewage for HPV-3 and HPV-10 (72).

Clinical surveillance of HPV infections in Canada has notable gaps and limitations. It is difficult to implement surveillance for HPV, in part because the vast majority of HPV infections are asymptomatic (65, 90). For cutaneous and low-risk HPVs, clinicians focus heavily on treating the symptoms rather than type identification, as treatments are generally similar regardless (61). Even though HPV testing is available in many regions of Canada, accessibility is limited for underserviced populations, including new immigrants (91), transgender men (91), Indigenous people (12), and those living in northern, remote, and isolated communities (12), resulting in lower rates of cervical cancer screening and higher rates of cervical cancer (12, 92–95). Although presently there is no centralized reporting structure for clinical HPV testing in Canada, intergovernmental collaborations aim to implement a database and registry for monitoring infections, disease outcomes, and vaccination uptake (96). This tool could also prove valuable in the future for efficiently identifying new correlations between HPV types of unknown risk and their potential links to cancer.

The majority of clinical HPV tests in Canada do not discriminate between specific types, simply identifying whether a person is infected with HR-HPV (97, 98). Additionally, type-specific tests generally do not include all of the probable HR-HPV types; for example, HPV-67, HPV-69, and HPV-73 are not captured by routine clinical testing, but all were detected consistently in urban and town sites during this study. Furthermore, clinical testing is usually only available to biological females above 30 years of age, who have an increased risk of progression to cervical cancer (99). Although an effective strategy for cancer screening, it does little to characterize HPV in people under 25 years of age, who are the most likely demographic to have HPV (100). WBT fills these surveillance gaps, encompassing the entire population, including biological males who are rarely tested (99, 101). Vaccination against HPV was approved and expanded to biological males only recently (99), and the impacts of these vaccination campaigns could be monitored via wastewater testing.

Finally, clinical HPV testing is primarily conducted on cervical samples, but HPV infections extend beyond the cervix and are also linked to anal, penile, and oropharyngeal cancers. The ability to detect HPV in stool and urine offers a unique opportunity to detect shedding from individuals with urogenital and rectal infections, particularly among men. Research also suggests the virus may traverse the digestive tract, potentially enabling surveillance of oropharyngeal infections (72, 102). A broader understanding of circulating HPV types can help refine clinical testing protocols and guide the development and prioritization of future vaccines. Wastewater testing serves as a complementary tool to clinical screening, particularly for HPV-associated cancers that currently lack routine diagnostic testing in men.

We recognize that building-level wastewater surveillance, particularly in a correctional facility, entails unique ethical responsibilities. Because inmates and staff cannot opt out of this surveillance, and the data pertain to a defined population, care must be taken to protect privacy and prevent misuse of the findings. In our study, facilities are not identified by name, and results are interpreted only at a population level. Even though wastewater data cannot be traced to individuals, the perception of these data is a concern; for instance, mismanaged data might lead to negative perceptions. We emphasize that the goal of WBE in such settings should always be to benefit public health—for example, by enabling infection monitoring and prevention in the facility—while minimizing any risk of unfair scrutiny on the population.

A limitation of this study is that we failed to amplify three of the positive control plasmids (Fig. 1), suggesting that this method cannot amplify all relevant HPV types. The PCR assay utilized in this study targets a 151 bp region of the L1 gene that has been used for PCR amplification of multiple HPV types both independently and simultaneously since the 1990s (103). These generalized primers, termed the GP5/GP6+ primers, allow for the detection of a broad range of HPV types using PCR under a low annealing temperature, which enables mismatch acceptance and amplification of several types (77). Conveniently, a hypervariable region exists upstream of the GP5+ primers that enable genotyping (103–105). Experimental analysis has shown that PCR efficiency is not affected by three mismatches or less under these conditions (103). The plasmids that failed to amplify, HPV 39, HPV 51, and HPV 68, are known to have lower affinity for these primers as compared to other HR-HPV types due to greater than three mismatches in the primer binding regions (77, 104, 106). To address inefficiencies in amplification, future experiments could explore using a lower annealing temperature, incorporating additional primer sets, or touchdown PCR to increase sensitivity against all HPV types. The absence of particular HPV types in our data does not necessarily indicate their absence in wastewater or the general population.

In the context of wastewater, HPV DNA is likely derived from a complex mixture of lysed epithelial cells, partially degraded virions, or environmental degradation products. It cannot be definitively determined from this work to what extent signals are derived from HPV sequences integrated in host genomes; however, evidence from the literature would suggest the contribution would be minimal. While the integration of HR-HPV types into the host genome is a hallmark of carcinogenesis, integration is not required for viral replication, and HPV DNA often exists in an episomal (non-integrated) form during normal infection. Notably, during integration events, the HPV genome is often fragmented, and regions such as L1 are disrupted or deleted (107, 108). Additionally, it would be likely that the amount of actively reproducing virus and viral particles would far exceed the number of copies integrated into host genomes, with self-collected vaginal swab samples recording viral loads >10^10^ viral particles (109).

Another potential limitation of this study is the lack of direct clinical surveillance data, which constrains our ability to fully validate how wastewater data reflects community-level HPV prevalence. As HPV is not a reportable disease in Canada, comprehensive infection statistics are unavailable. However, some provincial-level data exist on HPV vaccine uptake. In Manitoba and Saskatchewan, 70% of school-age girls receive the full vaccine course, which falls below the national target, and uptake rates for school-age boys are not reported (110). The absence of individual-level infection data limits direct comparisons between wastewater and clinical prevalence, but future studies could leverage WBT in populations with varying vaccine coverage to assess its impact on community-wide HPV circulation. Additionally, as vaccinated cohorts continue to age, shifts in dominant HPV types may emerge, underscoring the value of wastewater surveillance for long-term monitoring.

In summary, this work demonstrates the feasibility of detecting HPV in wastewater and highlights its potential value in public health surveillance. Wastewater monitoring, used for tracking viruses like poliovirus and COVID-19, can similarly be applied to HPV, an important and underreported infection. Integrating HPV into routine wastewater surveillance could complement clinical testing by capturing broader population trends, including asymptomatic or unreported cases from institutional settings to large urban centers. This approach may also support the evaluation of vaccination efforts and reveal circulating high-risk genotypes, offering actionable insights to guide cancer prevention strategies. The ability to adapt wastewater surveillance to other pathogens hints toward a promising future for unbiased monitoring of community health in Canada and globally. These data will be important as we aim to protect communities against public health threats, both new and old.

## Data Availability

All FASTQ sequencing files used in this study have been deposited within the NCBI Sequence Read Archive (accession PRJNA1219107).

## SUPPLEMENTAL MATERIAL

Tables S1 and S2 aem.00348-25-s0001.pdf Table S1, reverse primer sequences used for each sample in this study; Table S2, summary of Illumina MiSeq sequencing results.

ASM does not own the copyrights to Supplemental Material that may be linked to, or accessed through, an article. The authors have granted ASM a non-exclusive, world-wide license to publish the Supplemental Material files. Please contact the corresponding author directly for reuse.
