# Supplementary material for: Identification of circulating human papillomavirus types through high-throughput sequencing of Canadian municipal and institutional wastewater samples
Source: Appl Environ Microbiol. 2025 Jun 5;91(7):e00348-25. doi: 10.1128/aem.00348-25 (PMC12285240; doi:10.1128/aem.00348-25)
Supplement: Tables S1 and S2 — Table S1, reverse primer sequences used for each sample in this study; Table S2, summary of Illumina MiSeq sequencing results. [file aem.00348-25-s0001.pdf]

Supplemental Table 1. Reverse Primer sequences used for each sample in this study. Each primer sequence includes an Illumina i7 adapter, sequencing primer site, unique sample barcode, and GP6+ primer site.

Sample ID	Reverse Primer Sequence (5’→3’)
Correctional Facility 1 05-12-2023	CAAGCAGAAGACGGCATACGAGATGTCCGCGTGACTGGAGTTCAGACGTGTGCTCTTCCGATCTGAAAAATAAACTGTAAATCATATTC
Correctional Facility 1 05-19-2023	CAAGCAGAAGACGGCATACGAGATGTGAAAGTGACTGGAGTTCAGACGTGTGCTCTTCCGATCTGAAAAATAAACTGTAAATCATATTC
Correctional Facility 1 05-26-2023	CAAGCAGAAGACGGCATACGAGATGTGGCCGTGACTGGAGTTCAGACGTGTGCTCTTCCGATCTGAAAAATAAACTGTAAATCATATTC
Correctional Facility 1 06-02-2023	CAAGCAGAAGACGGCATACGAGATGTTTCGGTGACTGGAGTTCAGACGTGTGCTCTTCCGATCTGAAAAATAAACTGTAAATCATATTC
Correctional Facility 1 06-09-2023	CAAGCAGAAGACGGCATACGAGATGAGTGGGTGACTGGAGTTCAGACGTGTGCTCTTCCGATCTGAAAAATAAACTGTAAATCATATTC
Correctional Facility 1 06-26-2023	CAAGCAGAAGACGGCATACGAGATACTGATGTGACTGGAGTTCAGACGTGTGCTCTTCCGATCTGAAAAATAAACTGTAAATCATATTC
Correctional Facility 1 07-04-2023	CAAGCAGAAGACGGCATACGAGATATTCCTGTGACTGGAGTTCAGACGTGTGCTCTTCCGATCTGAAAAATAAACTGTAAATCATATTC
Correctional Facility 1 07-11-2023	CAAGCAGAAGACGGCATACGAGATCGGCCCGTGACTGGAGTTCAGACGTGTGCTCTTCCGATCTGAAAAATAAACTGTAAATCATATTC
Correctional Facility 2 05-09-2023	CAAGCAGAAGACGGCATACGAGATCGTACGGTGACTGGAGTTCAGACGTGTGCTCTTCCGATCTGAAAAATAAACTGTAAATCATATTC
Correctional Facility 2 05-17-2023	CAAGCAGAAGACGGCATACGAGATGAGTGGGTGACTGGAGTTCAGACGTGTGCTCTTCCGATCTGAAAAATAAACTGTAAATCATATTC
Correctional Facility 2 05-23-2023	CAAGCAGAAGACGGCATACGAGATACTGATGTGACTGGAGTTCAGACGTGTGCTCTTCCGATCTGAAAAATAAACTGTAAATCATATTC
Correctional Facility 2 05-30-2023	CAAGCAGAAGACGGCATACGAGATTCTCGAGTGACTGGAGTTCAGACGTGTGCTCTTCCGATCTGAAAAATAAACTGTAAATCATATTC

Correctional Facility 2 06-06-2023	CAAGCAGAAGACGGCATACGAGATTAGCTTGTGACTGGAG TTCAGACGTGTGCTCTTCCGATCTGAAAAATAAACTGTAAA TCATATTC
Correctional Facility 2 06-08-2023	CAAGCAGAAGACGGCATACGAGATAGTTCCGTGACTGGAG TTCAGACGTGTGCTCTTCCGATCTGAAAAATAAACTGTAAA TCATATTC
Correctional Facility 2 06-13-2023	CAAGCAGAAGACGGCATACGAGATATGTCAGTGACTGGAG TTCAGACGTGTGCTCTTCCGATCTGAAAAATAAACTGTAAA TCATATTC
Correctional Facility 2 06-20-2023	CAAGCAGAAGACGGCATACGAGATCCGTCCGTGACTGGAG TTCAGACGTGTGCTCTTCCGATCTGAAAAATAAACTGTAAA TCATATTC
Correctional Facility 2 07-06-2023	CAAGCAGAAGACGGCATACGAGATGTCCGCGTGACTGGAG TTCAGACGTGTGCTCTTCCGATCTGAAAAATAAACTGTAAA TCATATTC
Correctional Facility 2 07-11-2023	CAAGCAGAAGACGGCATACGAGATGTGAAAGTGACTGGAG TTCAGACGTGTGCTCTTCCGATCTGAAAAATAAACTGTAAA TCATATTC
Correctional Facility 3 03-31-2023	CAAGCAGAAGACGGCATACGAGATCCGTCCGTGACTGGAG TTCAGACGTGTGCTCTTCCGATCTGAAAAATAAACTGTAAA TCATATTC
Correctional Facility 3 04-07-2023	CAAGCAGAAGACGGCATACGAGATAGTTCCGTGACTGGAG TTCAGACGTGTGCTCTTCCGATCTGAAAAATAAACTGTAAA TCATATTC
Correctional Facility 3 04-14-2023	CAAGCAGAAGACGGCATACGAGATATGTCAGTGACTGGAG TTCAGACGTGTGCTCTTCCGATCTGAAAAATAAACTGTAAA TCATATTC
Correctional Facility 3 05-15-2023	CAAGCAGAAGACGGCATACGAGATGGCTACGTGACTGGAG TTCAGACGTGTGCTCTTCCGATCTGAAAAATAAACTGTAAA TCATATTC
Correctional Facility 3 05-22-2023	CAAGCAGAAGACGGCATACGAGATCTTGTAGTGACTGGAG TTCAGACGTGTGCTCTTCCGATCTGAAAAATAAACTGTAAA TCATATTC
Correctional Facility 3 05-29-2023	CAAGCAGAAGACGGCATACGAGATAGTCAAGTGACTGGAG TTCAGACGTGTGCTCTTCCGATCTGAAAAATAAACTGTAAA TCATATTC
Correctional Facility 3 06-05-2023	CAAGCAGAAGACGGCATACGAGATGATCAGGTGACTGGAG TTCAGACGTGTGCTCTTCCGATCTGAAAAATAAACTGTAAA TCATATTC
Correctional Facility 3 06-09-2023	CAAGCAGAAGACGGCATACGAGATGTGGCCGTGACTGGAG TTCAGACGTGTGCTCTTCCGATCTGAAAAATAAACTGTAAA TCATATTC
Correctional Facility 3 06-19-2023	CAAGCAGAAGACGGCATACGAGATGTTTCGGTGACTGGAG TTCAGACGTGTGCTCTTCCGATCTGAAAAATAAACTGTAAA TCATATTC

Correctional Facility 3 06-26-2023	CAAGCAGAAGACGGCATACGAGATCGTACGGTGACTGGAG TTCAGACGTGTGCTCTTCCGATCTGAAAAATAAACTGTAAA TCATATTC
Regina 03-20-2023	CAAGCAGAAGACGGCATACGAGATCTTGTAGTGACTGGAG TTCAGACGTGTGCTCTTCCGATCTGAAAAATAAACTGTAAA TCATATTC
Regina 03-28-2023	CAAGCAGAAGACGGCATACGAGATGTGAAAGTGACTGGAG TTCAGACGTGTGCTCTTCCGATCTGAAAAATAAACTGTAAA TCATATTC
Regina 04-04-2023	CAAGCAGAAGACGGCATACGAGATGTGGCCGTGACTGGAG TTCAGACGTGTGCTCTTCCGATCTGAAAAATAAACTGTAAA TCATATTC
Regina 04-11-2023	CAAGCAGAAGACGGCATACGAGATGTTTCGGTGACTGGAG TTCAGACGTGTGCTCTTCCGATCTGAAAAATAAACTGTAAA TCATATTC
Regina 04-18-2023	CAAGCAGAAGACGGCATACGAGATTACCGTGTGACTGGAG TTCAGACGTGTGCTCTTCCGATCTGAAAAATAAACTGTAAA TCATATTC
Regina 04-25-2023	CAAGCAGAAGACGGCATACGAGATGACTCAGTGACTGGAG TTCAGACGTGTGCTCTTCCGATCTGAAAAATAAACTGTAAA TCATATTC
Regina 05-02-2023	CAAGCAGAAGACGGCATACGAGATGTAGGTGTGACTGGAG TTCAGACGTGTGCTCTTCCGATCTGAAAAATAAACTGTAAA TCATATTC
Regina 05-14-2023	CAAGCAGAAGACGGCATACGAGATCACAGTGTGACTGGAG TTCAGACGTGTGCTCTTCCGATCTGAAAAATAAACTGTAAA TCATATTC
Regina 05-23-2023	CAAGCAGAAGACGGCATACGAGATTCAGAGGTGACTGGAG TTCAGACGTGTGCTCTTCCGATCTGAAAAATAAACTGTAAA TCATATTC
Regina 05-30-2023	CAAGCAGAAGACGGCATACGAGATGACATGGTGACTGGAG TTCAGACGTGTGCTCTTCCGATCTGAAAAATAAACTGTAAA TCATATTC
Regina 06-06-2023	CAAGCAGAAGACGGCATACGAGATACTGTTGTGACTGGAG TTCAGACGTGTGCTCTTCCGATCTGAAAAATAAACTGTAAA TCATATTC
Regina 06-11-2023	CAAGCAGAAGACGGCATACGAGATATGAACGTGACTGGAG TTCAGACGTGTGCTCTTCCGATCTGAAAAATAAACTGTAAA TCATATTC
Regina 06-20-2023	CAAGCAGAAGACGGCATACGAGATCGGCACGTGACTGGAG TTCAGACGTGTGCTCTTCCGATCTGAAAAATAAACTGTAAA TCATATTC
Regina 07-04-2023	CAAGCAGAAGACGGCATACGAGATTCAAGGGTGACTGGAG TTCAGACGTGTGCTCTTCCGATCTGAAAAATAAACTGTAAA TCATATTC

Regina<br>07-11-2023	CAAGCAGAAGACGGCATACGAGATAGTTTGGTGACTGGAGTTCAGACGTGTGCTCTTCCGATCTGAAAAATAAACTGTAAATCATATTC
Town Site 1<br>03-27-2023	CAAGCAGAAGACGGCATACGAGATAGTTCCGTGACTGGAGTTCAGACGTGTGCTCTTCCGATCTGAAAAATAAACTGTAAATCATATTC
Town Site 1<br>04-04-2023	CAAGCAGAAGACGGCATACGAGATACTGATGTGACTGGAGTTCAGACGTGTGCTCTTCCGATCTGAAAAATAAACTGTAAATCATATTC
Town Site 1<br>04-11-2023	CAAGCAGAAGACGGCATACGAGATATTCCTGTGACTGGAGTTCAGACGTGTGCTCTTCCGATCTGAAAAATAAACTGTAAATCATATTC
Town Site 1<br>04-18-2023	CAAGCAGAAGACGGCATACGAGATAAGAGTGTGACTGGAGTTCAGACGTGTGCTCTTCCGATCTGAAAAATAAACTGTAAATCATATTC
Town Site 1<br>04-25-2023	CAAGCAGAAGACGGCATACGAGATCTGTAGGTGACTGGAGTTCAGACGTGTGCTCTTCCGATCTGAAAAATAAACTGTAAATCATATTC
Town Site 1<br>05-02-2023	CAAGCAGAAGACGGCATACGAGATTCCAAGGTGACTGGAGTTCAGACGTGTGCTCTTCCGATCTGAAAAATAAACTGTAAATCATATTC
Town Site 1<br>05-14-2023	CAAGCAGAAGACGGCATACGAGATATGATAGTGACTGGAGTTCAGACGTGTGCTCTTCCGATCTGAAAAATAAACTGTAAATCATATTC
Town Site 1<br>05-23-2023	CAAGCAGAAGACGGCATACGAGATATATAGGTGACTGGAGTTCAGACGTGTGCTCTTCCGATCTGAAAAATAAACTGTAAATCATATTC
Town Site 1<br>05-30-2023	CAAGCAGAAGACGGCATACGAGATTTCGGTGTGACTGGAGTTCAGACGTGTGCTCTTCCGATCTGAAAAATAAACTGTAAATCATATTC
Town Site 1<br>06-06-2023	CAAGCAGAAGACGGCATACGAGATCTGTAGGTGACTGGAGTTCAGACGTGTGCTCTTCCGATCTGAAAAATAAACTGTAAATCATATTC
Town Site 1<br>06-13-2023	CAAGCAGAAGACGGCATACGAGATTCCAAGGTGACTGGAGTTCAGACGTGTGCTCTTCCGATCTGAAAAATAAACTGTAAATCATATTC
Town Site 1<br>06-20-2023	CAAGCAGAAGACGGCATACGAGATATGATAGTGACTGGAGTTCAGACGTGTGCTCTTCCGATCTGAAAAATAAACTGTAAATCATATTC
Town Site 1<br>07-11-2023	CAAGCAGAAGACGGCATACGAGATATATAGGTGACTGGAGTTCAGACGTGTGCTCTTCCGATCTGAAAAATAAACTGTAAATCATATTC
Town Site 2<br>03-27-2023	CAAGCAGAAGACGGCATACGAGATACTGTTGTGACTGGAGTTCAGACGTGTGCTCTTCCGATCTGAAAAATAAACTGTAAATCATATTC

Town Site 2<br>04-08-2023	CAAGCAGAAGACGGCATACGAGATATGAACGTGACTGGAG<br>TTCAGACGTGTGCTCTTCCGATCTGAAAAATAAACTGTAAA<br>TCATATTC
Town Site 2<br>04-11-2023	CAAGCAGAAGACGGCATACGAGATGAGTGGGTGACTGGAG<br>TTCAGACGTGTGCTCTTCCGATCTGAAAAATAAACTGTAAA<br>TCATATTC
Town Site 2<br>04-18-2023	CAAGCAGAAGACGGCATACGAGATCGGCACGTGACTGGAG<br>TTCAGACGTGTGCTCTTCCGATCTGAAAAATAAACTGTAAA<br>TCATATTC
Town Site 2<br>04-25-2023	CAAGCAGAAGACGGCATACGAGATTCAAGGGTGACTGGAG<br>TTCAGACGTGTGCTCTTCCGATCTGAAAAATAAACTGTAAA<br>TCATATTC
Town Site 2<br>05-01-2023	CAAGCAGAAGACGGCATACGAGATAGTTTGGTGACTGGAG<br>TTCAGACGTGTGCTCTTCCGATCTGAAAAATAAACTGTAAA<br>TCATATTC
Town Site 2<br>05-08-2023	CAAGCAGAAGACGGCATACGAGATTCTCGAGTGACTGGAG<br>TTCAGACGTGTGCTCTTCCGATCTGAAAAATAAACTGTAAA<br>TCATATTC
Town Site 2<br>05-24-2023	CAAGCAGAAGACGGCATACGAGATTACCGTGTGACTGGAG<br>TTCAGACGTGTGCTCTTCCGATCTGAAAAATAAACTGTAAA<br>TCATATTC
Town Site 2<br>05-30-2023	CAAGCAGAAGACGGCATACGAGATGACTCAGTGACTGGAG<br>TTCAGACGTGTGCTCTTCCGATCTGAAAAATAAACTGTAAA<br>TCATATTC
Town Site 2<br>06-21-2023	CAAGCAGAAGACGGCATACGAGATCACAGTGTGACTGGAG<br>TTCAGACGTGTGCTCTTCCGATCTGAAAAATAAACTGTAAA<br>TCATATTC
Town Site 2<br>07-05-2023	CAAGCAGAAGACGGCATACGAGATTCAGAGGTGACTGGAG<br>TTCAGACGTGTGCTCTTCCGATCTGAAAAATAAACTGTAAA<br>TCATATTC
Town Site 2<br>07-11-2023	CAAGCAGAAGACGGCATACGAGATGACATGGTGACTGGAG<br>TTCAGACGTGTGCTCTTCCGATCTGAAAAATAAACTGTAAA<br>TCATATTC
Winnipeg South<br>03-23-2023	CAAGCAGAAGACGGCATACGAGATGGCTACGTGACTGGAG<br>TTCAGACGTGTGCTCTTCCGATCTGAAAAATAAACTGTAAA<br>TCATATTC
Winnipeg South<br>03-29-2023	CAAGCAGAAGACGGCATACGAGATATGTCAGTGACTGGAG<br>TTCAGACGTGTGCTCTTCCGATCTGAAAAATAAACTGTAAA<br>TCATATTC
Winnipeg South<br>04-06-2023	CAAGCAGAAGACGGCATACGAGATTACTCAGTGACTGGAG<br>TTCAGACGTGTGCTCTTCCGATCTGAAAAATAAACTGTAAA<br>TCATATTC
Winnipeg South<br>04-12-2023	CAAGCAGAAGACGGCATACGAGATGTCCGCGTGACTGGAG<br>TTCAGACGTGTGCTCTTCCGATCTGAAAAATAAACTGTAAA<br>TCATATTC

Winnipeg South 04-17-2023	CAAGCAGAAGACGGCATACGAGATACCATTGTGACTGGAGTTCAGACGTGTGCTCTTCCGATCTGAAAAATAAACTGTAAATCATATTC
Winnipeg South 04-24-2023	CAAGCAGAAGACGGCATACGAGATTGGTTCGTGACTGGAGTTCAGACGTGTGCTCTTCCGATCTGAAAAATAAACTGTAAATCATATTC
Winnipeg South 05-01-2023	CAAGCAGAAGACGGCATACGAGATGGTGCGGTGACTGGAGTTCAGACGTGTGCTCTTCCGATCTGAAAAATAAACTGTAAATCATATTC
Winnipeg South 05-08-2023	CAAGCAGAAGACGGCATACGAGATTGGCACGTGACTGGAGTTCAGACGTGTGCTCTTCCGATCTGAAAAATAAACTGTAAATCATATTC
Winnipeg South 05-18-2023	CAAGCAGAAGACGGCATACGAGATTCGGTAGTGACTGGAGTTCAGACGTGTGCTCTTCCGATCTGAAAAATAAACTGTAAATCATATTC
Winnipeg South 05-25-2023	CAAGCAGAAGACGGCATACGAGATTACTCAGTGACTGGAGTTCAGACGTGTGCTCTTCCGATCTGAAAAATAAACTGTAAATCATATTC
Winnipeg South 06-01-2023	CAAGCAGAAGACGGCATACGAGATACCATTGTGACTGGAGTTCAGACGTGTGCTCTTCCGATCTGAAAAATAAACTGTAAATCATATTC
Winnipeg South 06-05-2023	CAAGCAGAAGACGGCATACGAGATTCGACTGTGACTGGAGTTCAGACGTGTGCTCTTCCGATCTGAAAAATAAACTGTAAATCATATTC
Winnipeg South 06-19-2023	CAAGCAGAAGACGGCATACGAGATTGGTTCGTGACTGGAGTTCAGACGTGTGCTCTTCCGATCTGAAAAATAAACTGTAAATCATATTC
Winnipeg South 06-22-2023	CAAGCAGAAGACGGCATACGAGATGGTGCGGTGACTGGAGTTCAGACGTGTGCTCTTCCGATCTGAAAAATAAACTGTAAATCATATTC
Winnipeg South 06-29-2023	CAAGCAGAAGACGGCATACGAGATTGGCACGTGACTGGAGTTCAGACGTGTGCTCTTCCGATCTGAAAAATAAACTGTAAATCATATTC
Winnipeg South 07-10-2023	CAAGCAGAAGACGGCATACGAGATAAGAGTGTGACTGGAGTTCAGACGTGTGCTCTTCCGATCTGAAAAATAAACTGTAAATCATATTC

Supplementary Table 2. Summary of Illumina MiSeq Sequencing Results.

Sample ID	Reads generated	Reads passing quality control metrics	Reads matching HPV[1]
Correctional Facility 1 05-12-2023	953	680	608
Correctional Facility 1 05-19-2023	86419	84755	79175
Correctional Facility 1 05-26-2023	121316	6051	6
Correctional Facility 1 06-02-2023	82750	73120	72188
Correctional Facility 1 06-09-2023	160	38	0
Correctional Facility 1 06-26-2023	411	166	22
Correctional Facility 1 07-04-2023	13100	11817	11494
Correctional Facility 1 07-11-2023	44398	21415	17060
Correctional Facility 2 05-09-2023	8449	4953	2570
Correctional Facility 2 05-17-2023	5345	3425	3158
Correctional Facility 2 05-23-2023	45766	37218	35605
Correctional Facility 2 05-30-2023	352579	32564	32227
Correctional Facility 2 06-06-2023	19	8	3
Correctional Facility 2 06-08-2023	22545	20930	20774
Correctional Facility 2 06-13-2023	59255	55292	54996
Correctional Facility 2 06-20-2023	28208	26218	26023
Correctional Facility 2 07-06-2023	101281	2599	2215
Correctional Facility 2 07-11-2023	35395	32699	32263
Correctional Facility 3 03-31-2023	220	9	7
Correctional Facility 3 04-07-2023	147145	43217	20928
Correctional Facility 3 04-14-2023	60710	37091	29681
Correctional Facility 3 05-15-2023	66411	63846	62515
Correctional Facility 3 05-22-2023	549222	209306	74209
Correctional Facility 3 05-29-2023	27530	7333	1108
Correctional Facility 3 06-05-2023	11	3	1
Correctional Facility 3 06-09-2023	288878	259344	257336
Correctional Facility 3 06-19-2023	32635	30610	60471
Correctional Facility 3 06-26-2023	25427	23619	23491
Regina 03-20-2023	1604670	977406	940567
Regina 03-28-2023	85722	5723	5189
Regina 04-04-2023	1222865	980588	955491
Regina 04-11-2023	1384988	1256136	1214293
Regina 04-18-2023	78973	1062	915
Regina 04-25-2023	25133	5500	5426
Regina 05-02-2023	26855	12229	13973
Regina 05-14-2023	15593	8045	0
Regina 05-23-2023	114969	49029	48140
Regina 05-30-2023	122394	46454	39786

Regina 06-06-2023	34261	31349	31039
Regina 06-11-2023	17101	15859	15765
Regina 06-20-2023	7710	6737	6632
Regina 07-04-2023	1572	1338	1308
Regina 07-11-2023	50799	46806	46509
Town Site 1 03-27-2023	1447234	1275482	1184891
Town Site 1 04-04-2023	2176081	1217498	1058755
Town Site 1 04-11-2023	906088	545512	314641
Town Site 1 04-18-2023	892997	400714	96889
Town Site 1 04-25-2023	11278	5847	2800
Town Site 1 05-02-2023	34806	29883	29034
Town Site 1 05-14-2023	45758	39622	30778
Town Site 1 05-23-2023	53228	49704	48680
Town Site 1 05-30-2023	53878	26614	17789
Town Site 1 06-06-2023	259184	193588	165519
Town Site 1 06-13-2023	525481	486131	484088
Town Site 1 06-20-2023	503003	204896	189473
Town Site 1 07-11-2023	234100	216011	215072
Town Site 2 03-27-2023	341921	42546	34996
Town Site 2 04-04-2023	42705	36463	36209
Town Site 2 04-11-2023	35675	27794	26861
Town Site 2 04-18-2023	198216	16403	15522
Town Site 2 04-25-2023	167026	136946	135328
Town Site 2 05-01-2023	99116	87372	81496
Town Site 2 05-08-2023	27409	26293	26073
Town Site 2 05-24-2023	4679	4040	3969
Town Site 2 05-30-2023	645	385	347
Town Site 2 06-21-2023	56823	53747	53383
Town Site 2 07-05-2023	61198	57797	27459
Town Site 2 07-11-2023	15551	13374	13263
Winnipeg South 03-23-2023	5025	760	376
Winnipeg South 03-29-2023	1364466	1250640	1208711
Winnipeg South 04-06-2023	88800	178	28
Winnipeg South 04-12-2023	1459725	1155066	1044818
Winnipeg South 04-17-2023	30466	9868	8830
Winnipeg South 04-24-2023	24916	21031	20890
Winnipeg South 05-01-2023	34282	863	834
Winnipeg South 05-08-2023	42296	27365	26645
Winnipeg South 05-18-2023	14441	13401	13323
Winnipeg South 05-25-2023	9627	8837	875
Winnipeg South 06-01-2023	5434	810	295
Winnipeg South 06-05-2023	4154	3580	3517
Winnipeg South 06-19-2023	20466	18649	18477

Winnipeg South 06-22-2023	27943	25903	25756
Winnipeg South 06-29-2023	137675	117721	116700
Winnipeg South 07-10-2023	64946	54273	53439
Plasmid Type 16	29199	21328	21081
Plasmid Type 18	224660	164442	162952
Plasmid Type 31	66	9	7
Plasmid Type 33	359609	264691	262940
Plasmid Type 35	384578	276478	275706
Plasmid Type 39	47955	36	7
Plasmid Type 45	837033	632546	629784
Plasmid Type 51	839	69	65
Plasmid Type 52	52318	2098	2055
Plasmid Type 56	689957	509936	507688
Plasmid Type 58	302832	227255	225377
Plasmid Type 59	28887	19025	18828
Plasmid Type 66	28402	18683	18547
Plasmid Type 68	5179	3181	3
Negative Control	616	5	0

[1]Reads were considered to match HPV if the mapping region was 90 base pairs or more.
